# Epidemiology of survival pattern and its predictors among HIV positive patients on highly active antiretroviral therapy in Southern Ethiopia public health facilities: a retrospective cohort study

**DOI:** 10.1186/s12981-020-00307-x

**Published:** 2020-08-05

**Authors:** Abewa Kebede, Fasil Tessema, Gadisa Bekele, Zerihun Kura, Hailu Merga

**Affiliations:** 1 SNNP Health Bureau, Hawassa, Ethiopia; 2 Department of Epidemiology, Institute of Health, Jimma University, Jimma, Ethiopia; 3 School of Nursing, Institute of Health, Jimma University, Jimma, Ethiopia

**Keywords:** Epidemiology, Survival pattern, Predictor, Highly active, Antiretroviral therapy, Ethiopia

## Abstract

**Background:**

In resource poor countries like Ethiopia, little is known about the survival of patients treated with antiretroviral therapy which depends on different factors. Evidence shows that mortality has been high particularly in the first 3 to 6 months of initiating antiretroviral therapy. Hence, the study aimed to assess the Epidemiology of survival pattern and its determinants among adult HIV positive patients on highly active antiretroviral therapy.

**Methods:**

Retrospective cohort study was employed among a total of 455 records of patients who were enrolled on antiretroviral therapy from September 2006 to August 2010. Socio-demographic, clinical, immunological, behavioral, and date of antiretroviral treatment initiation including date of follow up status were extracted. Significant predictor variables were identified by fitting Cox’s proportional hazard model using a backward stepwise method and statistical significance variables were declared based on a p-value less than 0.05.

**Results:**

A total of 455 adult HIV/AIDS patients on ART contributed to 886.05-person-year of observation and 65.7% were alive and on treatment, 17.1% were lost to follow up and 7.5% died. The study showed that the estimated mortality was 4.4%, 5.3%, 6.1%, 7%, 7.5% and 7.5% at 6, 12, 24, 36, 48 and 60 months of follow up period, respectively. The overall incidence rate of mortality was 4.2 per 100 person-years of observation. In multivariate analysis age 45 and above (AHR: 3.72, 95% CI 1.21–11.4), bedridden functional status (AHR: 17.4, 95% CI 6.21–48.79), poor ART drug adherence (AHR: 4.52,95% CI 2.05–9.96), Tuberculosis co-infection (AHR: 4.1, 95% CI 1.84–9.13), non-disclosure (AHR: 4.9, 95% CI 1.82–12.89) and severe anemia (AHR: 5.1, 95% CI 1.81–14.21) were found predictors.

**Conclusion:**

Patients with older age, tuberculosis infection, bedridden patients and severe anemia were predictors. Tracing poorly adhered patients and giving drug counseling as well as encouraging them for disclosure to their families is crucial to improve their survival.

## Background

Survival time for infected Human immune virus (HIV) patients is the survival of HIV patients from the date of initiation of antiretroviral therapy (ART) to either death, loss to follow-up, transfer to other health institutions or to live follow-up [1]. Globally, about 36.9 million people live with HIV, of whom 35.1 million are adults in 2017, and more than half of them had access to antiretroviral therapy (ART), which significantly improved the survival of HIV patients. As a result, high global efforts have been made to increase ART coverage, and access to ART has also been improved in both developed and developing countries [2, 3].

In developing countries especially in sub-Saharan Africa, through further decentralization, treatment coverage can hopefully continue to improve patients in care. However, due to co-infections, early mortality remains high, especially among individuals with advanced diseases [2, 4]. Evidence shows that Eastern and Southern Africa remains the most affected region, accounting for 45% of the world’s HIV infections and 53% of people living with HIV. However, although evidence has shown good coverage of treatment among women, there are huge challenges to gender inequalities and gender-based violence combined with physiological factors. Although HIV care and treatment services are decentralized to selected health centers to reduce morbidity and mortality, the specific nature of disease demographics in poor resources, with a particularly high prevalence of TB, HBV, malnutrition and other bacterial infections, would have an impact on the nature of disease response that ultimately changes the survival pattern [3, 5].

Even though access to ARV therapy has shown significant clinical improvement by achieving the goal of therapy, many early deaths are associated with socio-demographic, behavioral risk and health factors [6–8]. Two years of follow-up study from India showed that 12.13% were lost to follow-up, 31.1% were transferred to other health facilities and 40.6% were alive. The finding indicated that the majority of deaths occurred during the first 6 months of therapy due to factors such as age, gender, hemoglobin and body weight [9]. Evidence shows that the early mortality rate in sub-Saharan Africa varies from 8 to 26%, with the majority of deaths occurring in the first few months [10]. Similarly, findings from Ghana indicated that about 44.4% of deaths occurred prior to the first month of initiation of ART and the WHO clinical stage, CD4 counts and hemoglobin levels were identified as indicators of disease progression [11].

Ethiopia has adopted a test-and-treat offering ART to all PLHIVs regardless of CD4 count, enabling more PLHIVs to have access to ART that can further reduce morbidity and mortality as well as the number of new infections. Approximately 610,000 people are living with HIV in Ethiopia in 2017, with approximately 15,000 deaths/year [3, 4, 12]. Findings from Dilla Hospital, Ethiopia, among adult cohorts of ART patients showed that the median follow-up period was 25 months and 77.7% were alive with survival patterns and continued hospital treatment, 9.2% were reported dead, 8% were transferred, and 5.1% lost follow-up [13]. Findings from previous HIV-infected person mortality studies show that the baseline level of hemoglobin, gender and adherence to ART were important factors for HIV survival. The high rate of loss to follow-up, poor CD4 recovery and poor body weight improvement after 6 months of follow-up is one of the determining problems associated with adherence and possible treatment failure [14, 15].

Although there has been an increasing interest among researchers in determining factors that influence the survival of people in HAART, there is no evidence available to identify determinants of mortality in patients receiving ART care and support services in public health facilities in the study area. The aim of this study was therefore to assess the Epidemiology of survival pattern and its determinants among adult HIV-positive patients on highly active antiretroviral therapy in public health facilities in Sawla Town, Southern Ethiopia.

## Methods

### Study design and setting

A retrospective cohort study was conducted from March 01, 2019, to April 10, 2019, in two public health facilities for patients who were started ART from September 2013 to August 2018. These health facilities are Sawla General Hospital and Sawla Health Center located in Goffa Zone, Southern Nation Nationalities People Regional State in southern Ethiopia. The town is located 285 km from Hawassa and 515 km from Addis Ababa, the capital of Ethiopia, in Southern direction. These two health facilities serve more than 1 million people from the surrounding. Since the establishment of Hospital (2002) and Health center (1998), a total of 920 (394 on treatment during 2011) patients started ART in Hospital and a total of 1094 (519 on treatment during 2011) patients started ART in Health center.

### Population and sampling

The study population consisted of all randomly selected adult HIV positive individuals (15 years of age and above) who started HAART between September 2013 to August 2018 in Sawla General Hospital and Sawla health center. HIV positive adults aged 15 and above who started ART with complete baseline data and intake form were included in the study. But, HIV-positive individual records having incomplete baseline data were excluded from the analysis. Among 467 electronic patient records reviewed, 308 were alive, 80 lost, 45 transferred out and 34 died. Totally, 8 with incomplete electronic records (5 among alive, 2 among lost and 1 among transferred out) and 4 patients who started ART since August 2010 were excluded from the study. The sample size was calculated by using sample size estimate for Longitudinal Studies with 95% confidence level, 80% power, one exposed to one non-exposed allocation ratio and the event of interest in exposed (WHO clinical stage III and IV) and non-exposed group (WHO clinical stage I and II) [16]. Then, the final sample size after the addition of 10% as a contingency for incompleteness, the total sample size was 467. To select study subjects, simple random sampling technique was employed by using Excel commands (Microsoft office add-ins and data analysis toolpak). All electronic records of patients started ART from September 2013 to August 2018 in each facility were listed in a separate Excel spreadsheet by using a unique ART number to prepare the sampling frame. Then 467 records were selected randomly. Finally, selected unique ART numbers were used to extract data from the electronic database.

### Measurements

HAART-initiated patients were followed until the date of death, loss to follow-up, transferring out, or completion of the study. Individuals who were on HAART, lost to follow-up, or had transferred out at the end of the study period were censored; that is, they were considered to be alive for the time that they had been under follow-up. The outcome event studied was survival and that censoring occurred for loss to follow up, transfer or study completion. The survival time was calculated in months using the time between the dates of treatment initiation and the date of the event (death) or date of censoring. Drug adherence was assessed in the study patient medication diary from the record. Then classification was done based on WHO classification and poor adherence was defined if the percentage of the missed dose was between < 85% (> 6 doses of 30 doses or > 9 doses of 60 doses) as documented by ART physician, fair adherence was defined as if the percentage of the missed dose was between 85 and 94% (3–5 doses of 30 doses or 3–9 dose of 60 doses) as documented by ART physician, and Good Adherence was defined as if the percentage of missed dose is between > 95% (< 2 doses of 30 doses or < 3 dose of 60 doses) as documented by ART physician. Substance use in this study was referred to as use of at least one of the substances commonly known in Ethiopia (alcohol, khat, tobacco, cannabis, cocaine and heroin). In our study TB co-infection was assessed by taking clinical lab data.

### Data collection procedure

The electronic database format which was developed by the Communicable Disease Control (CDC) was used as a data extraction tool. Secondary data routinely collected for clinical monitoring and evaluation purposes in the health facilities were used. Data was extracted retrospectively from all eligible electronic records of adult HIV/AIDS patients on ART from the ART database form. First, the profiles of all patients who started ART from September 2013 to August 2018 were evaluated and patients who started ART since August 2018 were excluded. Then, information about study participants, such as socio-demographic characteristics, clinical and immunological characteristics, behavioral characteristics, medication and prophylaxis, and survey endpoints were retrieved from the clinical records in the electronic database of HIV/AIDS patients by trained ART data clerks. Two ART data clerks who were trained on ART data management were recruited from the Hospital and Health center. In order to ensure the quality of collected data, one-day intensive training was given for data extractors about the objective and techniques of data extraction. After 1 day training, data extraction was started and different documents for the same patients were triangulated in case of odd values, non-logical data, or missed data.

### Statistical analysis

STROBE checklist was used to analyze and report data [17]. The Excel data were reshaped and merged and exported to Statistical Package for Social Sciences (SPSS) version 23 (IBM SPSS Statistics for Macintosh, Version 23.0 Armonk, NY) for analysis. After data cleaning by SPSS version 21, descriptive statistics for socio-demographic, baseline clinical, laboratory, and behavioral characteristics were done. Median and interquartile range (IQR) were calculated for baseline hemoglobin and CD4 count. Total Person years of observation were calculated for HIV positive patients during the study period. The Kaplan–Meier (KM) model was used to estimate the survival function. Cox proportional hazards model was used to identify independent factors associated with time to death. Factors associated with time to death at 25% significant level in the bivariate Cox proportional hazard model analysis were included in the final multivariate Cox proportional hazards model. Then, significant predictor variables were identified by fitting Cox’s proportional hazard model using a backward stepwise method. The results of the final model were expressed in terms of hazard ratio (HR) and 95% confidence intervals (CI) and statistical significance were declared based on p-value less than 0.05. The proportional hazards assumption has been checked using the log–log plot and plots of partial residuals against rank time.

## Results

### Socio-demographic characteristics of participants

A total of 467 HIV-positive individual records were reviewed. Of these, 455 (97.4%) had complete socio-demographic, baseline clinical, and immunological data. Out of 455 patients on HAART, more than half (58.3%) were females and the mean age was 31.5 (SD = 9.4) years. More than half, 60.4%, of the patients lived in urban areas. Nearly half (49.7%) of them were Orthodox Christian by their religion, about two-third (65.6%) of them were married and 38.7% had primary education (Table 1).

**Table 1:** Socio-demographic characteristics of the study subjects in public health facilities, Southern Ethiopia, 2018

Variables	Category	Follow up status				Total, N (%)
		Active n (%)	Lost n (%)	Transfer out n (%)	Dead, n (%)	
Sex	Male	104 (22.8)	41 (9)	24 (5.3)	21 (4.6)	190 (41.7)
	Female	195 (42.8)	37 (8.1)	20 (4.4)	13 (2.8)	265 (58.3)
Age	15–24	69 (15.2)	21 (4.6)	15 (3.3)	7 (1.52)	112 (25.1)
	25–34	133 (29.2)	32 (7.1)	13 (2.8)	10 (2.1)	188 (41.3)
	35–44	68 (14.9)	15 (3.3)	12 (2.6)	5 (1.1)	100 (22)
	≥ 45	29 (6.3)	10 (2.1)	4 (0.9)	12 (1.8)	55 (12.1)
Residence	Urban	182 (40)	44 (9.7)	30 (6.6)	19 (4.2)	275 (60.4)
	Rural	117 (25.7)	34 (7.5)	14 (3.1)	15 (3.3)	180 (39.6)
Educational level	Illiterate	95 (20.9)	28 (6.2)	7 (1.6)	12 (2.6)	142 (31.3)
	Primary	115 (25.3)	35 (7.7)	16 (3.5)	10 (2.2)	176 (38.7)
	Secondary	62 (13.6)	9 (2)	19 (4.2)	4 (0.9)	93 (20.5)
	Tertiary	27 (6)	6 (1.3)	2 (0.4)	8 (1.8)	43 (9.5)
Marital status	Never married	43 (9.5)	10 (2.2)	6 (1.3)	6 (1.3)	65 (14.3)
	Married	192 (42.2)	55 (12.1)	34 (7.5)	18 (4)	299 (65.6)
	separated	25 (5.5)	4 (0.9)	2 (0.4)	8 (1.8)	39 (8.6)
	divorced	20 (4.5)	5 (0.9)	1 (0.2)	2 (0.4)	28 (6)
	windowed	19 (4.2)	4 (0.9)	1 (0.2)	0	24 (5.3)
Occupation	Daily laborer	64 (14.1)	25 (5.5)	6 (1.3)	4 (0.9)	99 (21.8)
	Government employee	60 (13.2)	9 (2)	9 (2)	6 (1.3)	84 (18.46)
	Merchant	61 (13.4)	12 (2.6)	9 (2)	5 (1.1)	87 (19.1)
	NGO	3 (0.7)	1 (0.2)	6 (1.3)	0	10 (2.2)
	Farmer	47 (10.3)	16 (3.5)	8 (1.5)	7 (1.5)	87 (19.1)
	Driver	44 (9.7)	12 (2.6)	2 (0.4)	5 (1.1)	63 (13.8)
	Others	20 (4.4)	3 (0.7)	4 (0.9)	7 (1.5)	34 (7.5)

### Baseline clinical and laboratory characteristics of the study subjects

The mean weight at ART initiation was 53.09 kg [interquartile range (IQR, 46 kg–59 kg)]. The median hemoglobin level and CD4 counts were 11.6 mg/dL (IQR, 9.6–13.1) and 309 cells/mm^3^ (interquartile range IQR, 192–484) respectively. Concerning with preventive prophylaxis, more than three-fourth (78.7%) patients received cotrimoxazole preventive therapy (CPT) and about 77.6% patients received Isoniazid prophylaxis at the time of ART initiation. Out of 455 patients, 67 (14.7) patients had tuberculosis co-infection at the time of ART initiation and majority (41.1%) of them were in WHO stage I at the time of ART initiation. Our finding revealed that majority (84.4%) of them had good ART adherence and most of them (73.4%) initiated ART at CD4 ≥ 200 cells/μL (Table 2).

**Table 2:** shows baseline clinical and laboratory characteristics of the participants in public health facilities, Southern Ethiopia, 2018

Variables	Category of variables	Follow up status				Total n (%)
		Active n (%)	Lost for FU n (%)	Transfer out n (%)	Dead n (%)	
Weight	≤ 40	26 (5.7)	8 (1.8)	6 (1.3)	4 (1.1)	44 (9.7)
	40.1–50	107 (23.5)	14 (3.2)	12 (2.6)	14 (3.1)	147 (32.3)
	50.1–60	104 (22.8)	32 (7)	16 (3.5)	14 (3.1)	166 (36.5)
	> 60	62 (13.6)	24 (5.3)	10 (2.2)	2 (0.4)	98 (21.5)
CD4 count	≤ 100	23 (5.1)	8 (1.7)	9 (2)	10 (2.2)	50 (11.1)
	101–200	43 (9.4)	11 (2.4)	12 (2.6)	5 (1.1)	71 (15.6)
	> 200	233 (51.2)	59 (13)	23 (5.1)	19 (4.2)	334 (73.4)
Anemia	No anemia	159 (34.9)	27 (5.9)	13 (2.8)	13 (2.9)	212 (46.6)
	Mild	76 (16.7)	21 (4.6)	16 (3.5)	6 (1.3)	119 (26.2)
	Moderate	55 (12.1)	25 (5.5)	15 (3.3)	5 (1.1)	100 (22)
	Sever	9 (2)	5 (1.1)	0	10 (2.2)	29 (6.4)
BMI	≥ 18.5	219 (48.1)	56 (12.3)	20 (4.4)	17 (3.7)	312 (68.6)
	16–18.4	54 (11.9)	18 (4)	14 (3.1)	11 (2.4)	97 (21.3)
	< 16	26 (5.7)	4 (0.9)	10 (2.2)	6 (1.3)	46 (10.1)
INH Prophylaxis CPT	Yes	256 (56.3)	59 (13)	26 (5.7)	17 (3.7)	358 (78.7)
	No	43 (9.5)	19 (4.2)	18 (4)	17 (3.7)	97 (21.3)
	Yes	235 (51.6)	59 (13)	40 (8.8)	30 (6.6)	364 (80)
	No	64 (14.1)	19 (4.2)	4 (0.9)	4 (0.9)	91 (20)
WHO clinical staging	I	142 (31.2)	31 (6.8)	13 (2.8)	4 (0.9)	190 (41.7)
	II	60 (13.2)	19 (4.2)	7 (1.5)	7 (1.5)	93 (20.4)
	III	82 (18)	25 (5.5)	18 (4)	13 (2.9)	138 (30.5)
	IV	15 (3.3)	3 (0.7)	6 (1.3)	10 (2.2)	34 (7)
Adherence	Good	268 (58.9)	40 (8.8)	41 (9.1)	19 (4.2)	368 (80.9)
	Fair	16 (3.5)	6 (1.3)	0	0	22 (4.8)
	Poor	15 (3.3)	32 (7)	3 (0.7)	15 (3.3)	65 (14.3)
Functional status	Working	217 (47.7)	56 (12.3)	27 (5.9)	10 (2.2)	310 (68.1)
	Ambulatory	76 (16.7)	18 (3.9)	11 (2.4)	12 (2.6)	117 (25.7)
	Bedridden	6 (1.3)	4 (0.9)	6 (1.3)	12 (2.2)	28 (6.2)
Substance use	Yes	43 (9.4)	17 (3.73)	15 (3.29)	12 (2.63)	87 (19.1)
	No	256 (56.2)	61 (13.4)	29 (6.4)	22 (4.8)	368 (80.9)
Evidence of TB at start of ART	Yes	32 (7.1)	7 (1.5)	8 (1.5)	21 (4.6)	67 (14.7)
	No	267 (58.7)	71 (15.6)	37 (8.1)	13 (5.1)	388 (85.3)

### Survival pattern of the cohorts

Out of 455 cohorts of adults ART patients, 299 (64.9%) were alive and continued their treatment in the health facilities, 78 (17.4%) were lost follow up and 34 (7.5%) died. The estimated mortality rate was 4.4%, 5.3%, 6.1%, 7%, 7.5% and 7.5% at 6, 12, 24, 36, 48 and 60 months follow up period, respectively. Out of 34 deaths, 20 (58.8%) died within the first 6 months. The overall incidence rate of mortality during ART treatment was 4.2 per 100 person-year observations (PYOs).

### Predictors of mortality

Candidate variables that were associated with time to death at 25% significant level in the bivariate Cox regression analysis were sex, age, educational level, baseline weight, baseline BMI, baseline CD4 count, baseline hemoglobin, baseline WHO clinical staging, baseline functional status, baseline evidence of TB, tobacco smoking, alcohol drink, Khat chewing, disclosure status, ART adherence, CPT and INH prophylaxis. Then, candidate variables that were associated with time to death at 25% significant level in the bivariate Cox regression analysis were entered into multivariate Cox regression analysis. Predictor variables in multivariate Cox regression analysis were bedridden functional status, presence of tuberculosis at baseline, non-disclosure status, age 45 and above, severe anemia and poor ART drug adherence were statistically significant for death in HIV patients at P < 0.05 (Table 3).

**Table 3:** Multivariate Cox-regression analysis of the cohort studied in public health facilities, Southern Ethiopia, 2018

Variables	Category	Frequency		Bivariate CHR (CI)	Multivariate AHR (CI)
		Censored n (%)	Death n (%)		
Age	15–24	105 (24.2)	7 (1.5)	1	1
	25–34	178 (39.12)	10 (2.2)	0.8 (0.30–2.12)	0.98 (0.32–3.01)
	35–44	95 (20.9)	5 (1.1)	0.73 (0.23–2.32)	0.45 (0.13–1.57)
	≥ 45	43 (9.5)	12 (2.6)	4.53 (1.77–11.58)	3.72 (1.21–11.4)
Functional status	Working	300 (65.9)	10 (2.2)	1	1
	Ambulatory	105 (23.1)	12 (2.6)	3.24 (1.40–7.51)	1.11 (0.44–2.80)
	Bedridden	16 (3.5)	12 (2.6)	29.8 (12.54–71.1)	17.4 (6.2–48.79)
Anemia	No anemia	199 (43.7)	13 (2.9)	1	1
	Mild anemia	113 (24.8)	6 (1.3)	0.97 (0.37–2.57)	0.72 (0.23–2.23)
	Moderate anemia	95 (20.87)	5 (1.1)	1.03 (0.36–2.9)	2.48 (0.76–8.07)
	Severe anemia	14 (3.1)	10 (2.2)	13.79 (5.86–32.47)	5.07 (1.8–14.21)
Tuberculosis confection	Yes	46 (10.1)	21 (4.6)	11.05 (5.52–22.09)	4.10 (1.84–9.13)
	No	375 (82.4)	13 (2.9)	1	1
Adherence status	Good	371 (81.5)	13 (2.9)	1	1
	Poor	50 (11)	21 (4.6)	13.73 ( (6.8–27.61)	4.52 (2.05–9.96)
Disclosure status	Disclosed	349 (76.7)	7 (1.5)	1	1
	Not disclosed	72 (15.8)	27 (5.9)	12.81 (5.57–29.43)	4.9 (1.82–12.89)

*CHR* crude hazard ratio, *AHR* adjusted hazard ratio, *CI* confidence interval

## Discussion

The findings from the registered cohort showed that there were 34 (7.5%) deaths, providing an incidence density of mortality during ART treatment 4.2 per 100 person-year observations which was higher compared to other studies conducted in Ethiopia [18, 19] but lower than other studies conducted in Arbaminch, Ethiopia [20], Debremarkos Referral Hospital,

Northwest Ethiopia [21] and Cameroon [22] 16.7 and 52 per 100 person year observation (PYO) respectively.

This study showed that the majority of deaths were occurred in the first 6 months of ART initiation which was comparable to previous study [22]. The estimated mortality in the study period was 4.4%, 5.3%, 6.1%, 7%,7.5% and 7.5% at six, 12, 24, 36, 48 and 60 months of follow up period, respectively. This finding was comparable to the Nekemte referral hospital study [23].

Patients with poor ART adherence had the highest risk of death with 4.5 times more likely to die than adherent patients. Similarly study conducted in Ethiopia showed non-adherent participant’s had a mortality of 27 times compare to adherent ART patients [23]. Another comparable study from Ethiopia showed that the risks of death in non- adhered patients were 4 times higher compared to adhered patients [7]. The non-adherence to HAART leads to virologic, immunologic, clinical failure, and failure to suppress viral replication, thus increasing the likelihood of developing HIV mutations that could lead to the development of drug-resistant viral strains. Adherence to HAART is critical to the survival of HIV/AIDS infected people because poor adherence is the main reason for poor treatment outcomes among people receiving antiretroviral therapy [10, 24].

The mortality rate in this study was associated with baseline tuberculosis co-infection. People who started ART with tuberculosis co-infection were found to die almost 4 times higher than those who did not have baseline tuberculosis co-infection. These are explained by 67 HIV/AIDS patients with tuberculosis infection; 82% were in WHO clinical stage III and IV, 73% were ambulatory and bedridden, and 55% had CD4 counts of less than 100 cells/mm^3^ at baseline. This is consistent with studies in Ethiopia [13, 25] and India [26] which found that tuberculosis co-infection was a significant predictor of mortality. This may be because TB is the leading cause of death worldwide in HIV infection and mycobacterium tuberculosis is a virulent organism that can produce disease in HIV-infected individuals at any stage of the disease even when the immune suppression is minimal.

Patients who had severe anemia at baseline were 5 times higher risk of mortality than those who didn’t have. Similar studies done in Ethiopia [7, 18, 27] reported that low levels of hemoglobin at baseline are associated with high level of mortality among patients on HAART. Similar comparable finding have shown in Ethiopia that anemia could be an indicator of advanced disease or clinical features of some opportunistic infections which might aggravate the risk of death in ART patients [19].

Previous studies reported that the age of the patient was found to be the predictor of mortality, where the majority of patients in older age were more likely to die [28, 29]. Similar scenarios have happened in this study, age 45 years and above were nearly 4 times at higher risk of mortality than patients aged 15–24 years. This could be due to the fact that individuals are at higher risk of complications and respond poorly to ART as a result of the combined effect of aging, HIV infection, and antiretroviral treatment. It is known that as age increases immune status becomes incompetent which is considered to be a risk for many chronic diseases resulting in death.

This study found that patients with non-disclosure status were at high risk of death compared to patients with disclosure status. This finding is comparable to the study conducted in other parts of Ethiopia [16]. This may be due to the psychological readiness of the patients to receive social support from their partner in adapting the disease and taking the drug correctly. Consequently, non-disclosure has negative health impacts associated with distress, loneliness, and medical non-adherence as a means of hiding the presence of disease from others, and these factors may lead to a higher rate of mortality than those who have disclosed their HIV status.

A patient who initiated ART as bedridden (inability to attain self-care in the daily living) had the shortest survival rate than working (able to perform routine activities). Patients with bedridden functional status had 17.4 times higher risk of death compared with working patients which is similar with findings from Ethiopia [16, 30].

Although this study has strengths such as cross-checking of electronic database data with different documents at the time of non-logic and incomplete data, mortality may be underestimated due to loss of follow-up in patients likely to include more individuals dying at home without being reported.

## Conclusion

This study had identified independent significant predictors of survival in patients living with HIV/AIDS after the initiation of HAART. These factors include patients with older age, tuberculosis co-infection, bedridden functional status and severe anemia (hemoglobin less than 7 mg/dl). Hence, careful monitoring of patients, tracing poorly adhered patients and providing drug counseling and encouraging disclosure to their families is crucial to improve their survival. Moreover, special emphasis and closer follow-up should be given for HIV-TB co-infected patients.

## Data Availability

All data generated or analyzed during this study are included in this article

## Abbreviations

AIDS: Acquired immune deficiency syndrome
ART: Antiretroviral therapy
CD4: Cluster of differentiation 4
HAART: Highly active antiretroviral therapy
HIV: Human immune virus
MOH: Ministry of Health
WHO: World Health Organization
